# The Coming Operation for Cataract

**Published:** 1890-10

**Authors:** 


### THE COMING OPERATION FOR CATARACT.

Dr. T. J. Tyner, of Austin, has devised and performs an operation for cataract which is, as far as he knows, entirely original. It is ingenious, yet practical, and greatly simplifies the operation. It consists of a preliminary capsulotomy, which, of course, does away with iridectomy.

He prepared a paper on the subject, intending to read it at the recent International Medical Congress at Berlin, where he went as delegate of the Texas State Medical Association, but although his name and the title of the paper were on the programme for the first day's session in the Ophthalmological Section, and he was present and prepared to read it when his name was called, he did not have an opportunity to do so, for just as his name was called and he stepped forward with his paper, the chairman announced that the hour for adjournment had arrived, and adjourned the section. Dr. Tyner and many of his friends protested against the injustice, but without avail. The paper was also on the programme for the last day—but still opportunity was not given him to read it.

The above facts are submitted without comment.

But, truth is mighty and will prevail; the operation is a decided advance and—as surely as the sun shines—it will supersede all others for cataract extraction; it cannot be downed.

The *N. Y. Medical Journal*, of Sept. 20, publishes the paper in full, and that our readers may have the benefit of it, we will reproduce it in our next issue. Many European ophthalmologists who were attracted by the title of the paper, as it appeared on the programme, came to the author and asked him about his mode of operating, and all pronounced it ingenious and—so far as known to them—original. It is much to be regretted that the paper was not read and subsequently incorporated in the proceedings of the Congress.

